# Non-isoflavones Diet Incurred Metabolic Modifications Induced by Constipation in Rats via Targeting Gut Microbiota

**DOI:** 10.3389/fmicb.2018.03002

**Published:** 2018-12-04

**Authors:** Jialin Liu, Ruirui Chang, Xiaobei Zhang, Zhongzhao Wang, Jun Wen, Tingting Zhou

**Affiliations:** School of Pharmacy, Second Military Medical University, Shanghai, China

**Keywords:** isoflavones, constipation, intestinal microbiota, β-glucosidase, metabolism

## Abstract

Isoflavones, presenting in leguminous plants and the normal chow diet, are known to alter intestinal microbiota, yet their deficiency has not been widely studied for its effect on constipation in biochemical state of rats. Our previous study discovered the differences in pharmacokinetic traits of isoflavones from Semen *sojae praeparatum* fed with normal chow diet (ISO) and non-isoflavones diet (NISO). To gain insight into the key role of intestinal microbiota in constipation and metabolic differences caused by isoflavones deficiency, we observed a significant decrease in fecal pellet numbers, fecal water content, intestinal transit rate together with the serum concentrations of substance P (SP) and vasoactive intestinal peptide (VIP) in NISO group, compared with those in the ISO group. Following 16S rRNA compositional sequencing, results excluded the changes in intestinal microbiota over time and highlighted that a total of 5 phyla and 21 genera changed significantly, among which Firmicutes, Bacteroidetes, Blautia, Prevotella, Lactobacillus and Bifidobacterium were closely related to constipation. In addition, Lactobacillus, produceing β-glucosidase which contribute to biotransform glycosides into aglycons and exert the bioactivities consequently, was decreased after non-isoflavones diet intake. Meanwhile, predicted metagenomics indicated that the pathway of glycan biosynthesis and metabolism was markedly down-regulated after non-isoflavones diet intake. Taken together, the findings suggested that the changes in the dietary components could alter the biochemical state of rats, which may be triggered by the abnormal modifications facilitated by β-glucosidase-producing bacteria. Our study shed a new strategy to explore the relationship among disease phenotypes (D), intestinal microbiota (I), enzymes (E) and traits of metabolism (T) named as “DIET,” which can provide a reference for further study of the mechanism in regulation of intestinal bacteria-mediated diet on diseases.

## Introduction

The human gastrointestinal tract contains trillions of microbes, which is equivalent to more than 10-fold cells of human body (Maurice et al., 2013). Intestinal microbiota referred to as an essential organ of host plays a vital role in regulating metabolism, nutrition and immunity (Danneskiold-Samsøe et al., 2018). Recently, a great deal of interest has been paid toward the intimate relationship between intestinal microbiota and host health. Studies have indicated that abnormal gut microbiota is closely associated with various chronic diseases, including hypertension (Yan Q. et al., 2017), depression (Jiang et al., 2015), obesity (Del Chierico et al., 2018), metabolic syndrome (Haro et al., 2016) and some intestinal diseases (Casen et al., 2015; Thomas et al., 2016). Constipation, a common gastrointestinal disorder, is accompanied by defecating difficulty, abdominal pain and other discomforts, which significantly affects the quality of life (Somi et al., 2015). It can occur alone or in conjunction with other diseases, but only a few studies have elucidated the relationship between constipation and intestinal microbiota (Kang et al., 2015; Parthasarathy et al., 2017). The abundances of Bacteroidetes and Proteobacteria were increased, while the abundances of Firmicutes and Actinobacteria were decreased significantly in the mice gut microbiota of loperamide-induced constipation (Ren et al., 2017). In a clinical study, the microbiomes of the constipated obese adolescents exhibited decreased Prevotella and increased Blautia, Coprococcus, Ruminococcus and Faecalibacterium. Meanwhile, the abundances of Bifidobacterium, Lactobacillus and Streptococcus were increased in the gut of the constipated obese adolescents without statistical significance (Zhu et al., 2014). In daily life, medicine and diet are two main ways to alleviate constipation. Due to the side effects of medicine, developing diet that can improve constipation has become a mainstream trend. Among those factors that can alter intestinal microbiota, diet is particularly important as a main driver of microbial composition and metabolism in the gut, which contains many complex components, such as fiber, vitamins, Isoflavones, and so on. Different ingredients may have different effects on intestinal microbiota (Desai et al., 2016; Chen et al., 2018).

Isoflavones, phytoestrogens present in leguminous plants, can prevent cancer (Wang et al., 2016), osteoporosis (Kim et al., 2018) and cardiovascular diseases (Sathyapalan et al., 2018). In addition, they can also promote the development of small intestine and the growth of intestinal villi to alleviate constipation (Glabska et al., 2017). However, in soy and unfermented soy foods virtually all isoflavones exist in the form of glycosides which are characterized by their high hydrophilicity and inability to be absorbed by the intestine. Meanwhile, β-glucosidase produced by intestinal microbiota can hydrolyze the terminal non-reducing β-D-glucosidic bond to release β-glucose and associated ligand. Given the isoflavones structure, some reports indicated that glycosides are biotransformed into aglycones mainly by β-glucosidase in order to improve their bioavailabilities and exerted the bioactivities consequently (Chiou et al., 2014).

On account of isoflavones presenting in the normal chow diet of rats, our previous study compared the pharmacokinetic traits of isoflavones from Semen *sojae praeparatum* in rat plasma fed with two different diet and found that it increased the exposure of glycosides and decreased that of aglycones in the non-isoflavones diet group comparing with normal chow diet group (Yao et al., 2018). The findings suggested that the changes in the dietary components could alter the biochemical state of rats, which may be triggered by the abnormal modifications facilitated by β-glucosidase-producing bacteria. Due to the indispensable role of β-glucosidase in carbohydrate metabolism, it is very important to maintain its activity at a stable level. Most previous studies focused on the effects of single bacterial strain on glycosidic transformation and enzyme activity *in vitro*, which were unable to assess the uncultured bacteria in the intestinal tract (An et al., 2010). Moreover, the majority of researches aimed at the pathological state of the host, yet neglected the effects of substances on host physiological state (Nagata et al., 2016; Cross et al., 2017). Isoflavones are marketed as nutraceuticals, yet no study has investigated characteristics of the intestinal microbiota in healthy rats fed non-isoflavones diet by means of offsetting the impact of time.

Thus far, although a number of studies have demonstrated the association between disease phenotypes and intestinal microbiota or between enzymes and pharmacokinetic traits, too little is known about the internal mechanism between disease phenotypes and intestinal microbiota. In order to fill this gap in knowledge, high-throughput sequencing provide us technical support to explore the key role of intestinal microbiota in constipation and metabolic differences caused by non-isoflavones diet and to describe the interactions among disease phenotypes (D), intestinal microbiota (I), enzymes (E) and traits of metabolism (T) as “DIET.” Hence, we investigated the adverse effects of isoflavone deficiency on host health, compared the intestinal microbiota in non-isoflavones diet group with self-control method and predicted the changes of metabolic pathways after non-isoflavones diet intake. Ultimately, a sensitive strategy named DIET in our study was performed to find out: (1) how the non-isoflavones diet affects intestinal microbiota; (2) how the intestinal microbiota affects enzyme; (3) how the enzyme affects traits of metabolism; (4) how the traits of metabolism affects disease phenotypes.

## Materials and Methods

### Animals and Experimental Design

All animal protocols in this study were approved by the Ethics Committee of the Second Military Medical University (Shanghai, China). Twelve male Sprague-Dawley rats, weighing 180 ± 10g, were purchased from Experimental Animal Center of Second Military Medical University (Shanghai, China, SCXK < Hu > 2013-0016) and were housed in a standard room controlled the temperature (22 ± 2°C) and humidity (55 ± 5%) and illuminated for 12 h per day. After a week for adaptation, all animals were randomly divided into two groups on average: the control group and the experimental group. The rats of control group were fed with normal chow diet containing 0.038% isoflavones and these of experimental group were fed with non-isoflavones diet obtained from Trophic Animal Feed High-tech Co., Ltd. (Nantong, China) for 1 week. During the experimental period, they received water *ad libitum*. At the last day of adaptation (0 week) and the 7th day of diets intervention (1 week), the rats were individually transferred in cages and fresh fecal samples were collected and stored at -80°C for further analysis.

### Measurement of Defecation Status and Intestinal Transmission Function of Rats

At the 7th day of diets intervention (1 week), fecal samples of each rat were collected for 24 h to count and weigh. The water content was calculated as the percentage of wet weight occupied by the difference between wet and dry weights of fecal samples (Lee et al., 2017). After that, rats were fasted 12 h with water provided. Then, each rat was administered 1 mL of activated carbon suspension via gavage. 30 min later, the rats were killed by peritoneal anesthesia with 20% urethane solution and their blood and whole intestine were collected. In the absence of tension, the propulsive length of activated carbon and the full length of the intestine were measured. The percentage of propulsive length relative to full length of intestine was calculated to evaluate intestinal transmission function (Yan S. et al., 2017). After sitting at room temperature for 1 h, the serum was obtained by centrifugation at 3500 rpm for 20 min.

### Measurement of Serum Biochemical Indices

The concentrations of Substance P (SP) and Vasoactive Intestinal Peptide (VIP) in the serum samples were measured using rat SP and VIP ELISA kits (Mlbio, Shanghai, China), respectively.

### DNA Extraction and 16S rRNA Gene Sequencing

Total bacterial DNA was extracted from fecal samples using E.Z.N.A^®^ Mag-Bind^®^ Soil DNA Kit (Omega Bio-tek, Norcross, GA, United States) in accordance with the manufacturer’s instructions. Agarose gel electrophoresis was used to detect DNA integrity and concentration. Genomic DNA was accurately quantified by way of the Qubit 3.0 DNA Assay Kit (Life Technologies, CA, United States) to determine the amount of DNA that should be added to the PCR reaction. Primers 341F (CCTACGGGNGGCWGCAG), 805R (GACTACHVGGGTATCTAATCC) and specific barcode were established for PCR amplification targeting the V3-V4 hypervariable region of bacterial 16S rRNA gene. The reaction mixture included 15 μL of 2 × Taq master Mix (Vazyme Biotech, Nanjing, China), 1 μL of 10 μM Bar-PCR primer F, 1 μL of 10 μM primer R and 20 ng Genomic DNA, which added water to final volume of 30 μL. After amplification, PCR products were detected by agarose gel electrophoresis, purified with MagicPure Size Selection DNA Beads (Transgen Biotech, Beijing, China). Qubit 3.0 DNA Assay Kit (Life Technologies, CA, United States) was used to accurately quantify the recovered DNA, so as to apply Miseq platform (Illumina, San Diego, United States) for sequencing by Sangon Biotech Co., Ltd. (Shanghai, China) after equal mixing at 1:1 ratio. The DNA content of each sample was 10 ng when it was mixed equpcally, and the final sequencing concentration was 20 pmol.

### Bioinformatic Analysis

Raw fastq files were merged pair-end reads, cut primers, quality filtered and removed redundant using Usearch^1^ (version 10.0.240). All high-quality reads were clustered into operational taxonomic units (OTUs) by Unoise3 to create an OTU abundance table and then taxonomic information was assigned to representative OTUs based on Ribosomal Database Project (RDP) Classifier^2^ with a minimum confidence of 80%. Taking the sequence number in the sample with the least sequences as the standard of homogenization, the OTUs abundance information was normalized. Afterward, this normalized output data was utilized for subsequent analyses of alpha and beta diversity. Alpha diversity was evaluated by Shannon_e. Community richness was evaluated by Chao1. Furthermore, beta diversity applied UniFrac distances (weighted and unweighted) and Principal Coordinates Analyses (PCoA) to explain differences of samples. R software was a well tool to display beta diversity and volcano plot. Extended error bar in STAMP software was utilized to identify taxonomic changes with significant difference between two different groups. Prediction of functional genes needed to cluster sequence reads into OTUs based on Greengenes database (version 13.5) with 97% similarity cutoff. It could be used with online PICRUST^3^.

### Statistical Analysis

Body weight, food intake, fecal pellet numbers, fecal water content, intestinal transit rate together with the concentrations of SP and VIP and the ratio of Firmicutes to Bacteroidetes were presented as mean ± SEM and were analyzed with SPSS 21.0 software (IBM Inc., Chicago, United States). Student’s *t*-tests was used to analyze these data. Comparison of alpha diversity indexes among four groups was performed by Tukey HSD tests. Welch’s *t*-test was utilized to identify taxonomic changes with significant difference between two different groups in STAMP software. *P* < 0.05 was considered to be statistical significant.

## Results

### Isoflavones Deficiency Induces Symptoms of Constipation

**FIGURE 1:**
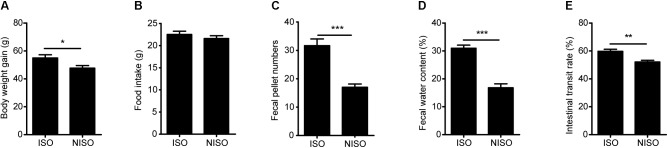
Effects of non-isoflavones diet treatment on the physical features of rats. **(A)** The body weight gain of non-isoflavones diet fed rats. **(B)** The food intake of rats fed with non-isoflavones diet. **(C)** Fecal pellet numbers of rats fed with non-isoflavones diet. **(D)** Fecal water content of rats fed non-isoflavones diet. **(E)** intestinal transit rate of rats fed with non-isoflavones diet. Data are presented as the mean ± SEM. ^∗^*P* < 0.05, ^∗∗^*P* < 0.01, ^∗∗∗^*P* < 0.001.

**FIGURE 2:**
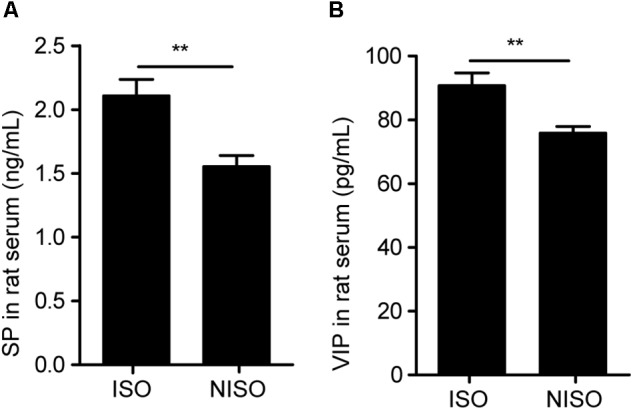
Non-isoflavones diet treatment significantly decreased serum parameters in rats. **(A)** Change in the SP level. **(B)** Change in the VIP level. SP, substance P. VIP, vasoactive intestinal peptide. Data are presented as the mean ± SEM. ^∗∗^*P* < 0.01.

Although the weight gain of rats in the experimental group (NISO, 1 week) was less than that of rats in the control group (ISO, 1 week) after 1 week of supplementation (P < 0.05; Figure 1A), there was no significant difference in food intake (Figure 1B). The constipation symptoms of rats in the experimental group were significantly exhibited. In terms of fecal pellet numbers, fecal water content and intestinal transit rate, there were significant difference between two groups. As shown in Figures 1C–E, the experimental group with non-isoflavones diet decreased the above three indicators compared with those in the control group. Additionally, the serum of experimental group displayed significantly lower concentrations of SP (P < 0.01; Figure 2A and Supplementary Table S1) and VIP (P < 0.01; Figure 2B and Supplementary Table S1) after 1 week of isoflavones deficiency. These findings indicated that the isoflavones deficiency could induce constipation.

### Diversity Analysis

All 24 fecal samples were successfully PCR amplified and sequenced. Targeting the V3-V4 hypervariable region of 16S rRNA, a total of 1,502,722 sequences were obtained from 24 samples collected at week 0 and 1. 1,502,361 high quality sequences were acquired after quality filtering. Sequences were clustered into 1501 unique OTUs.

To compare the differences of intestinal microbiota before and after isoflavones deficiency, alpha diversity indexes including Chao1 and Shannon_e were applied. The mean alpha diversity was lower in the control group after 1 week feeding (ISO, 1 week) than that before feeding (CISO, 0 week). Nevertheless, the difference in alpha diversity between these two groups could not be regarded as statistically significant. Treatment with non-isoflavones diet (NISO, 1 week) could significantly decrease intestinal microbiota diversity and richness on the basis of the above two indexes comparing with the experimental group before interference (CNISO, 0 week). The Chao1 index in the groups of CNISO and NISO were 1021.1 ± 49.72 and 725.9 ± 84.90 (*P* < 0.001), respectively. Shannon_e index in the groups of CNISO and NISO were 5.215 ± 0.029 and 4.495 ± 0.62 (*P* < 0.05), respectively.

**FIGURE 3:**
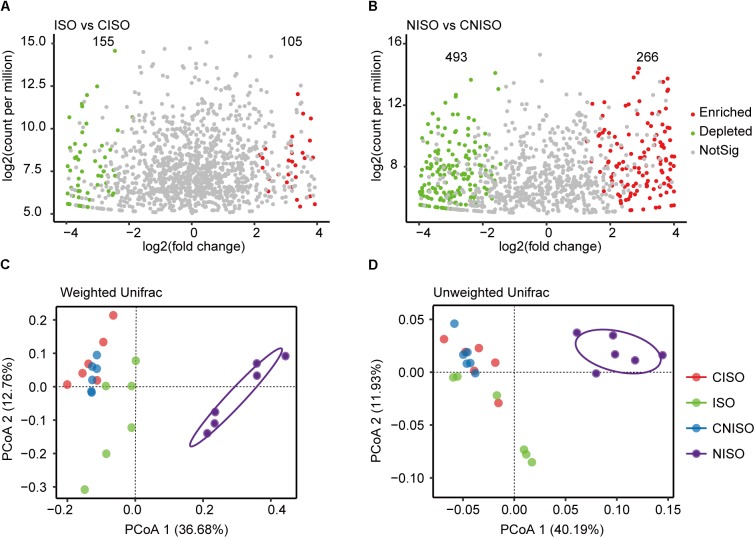
Comparison of intestinal microbiota structure among four different groups after two diets intervention for 1 week. Volcano plot showed the enriched and depleted OTUs. **(A)** Compared to CISO group, the changes was detected in ISO group. **(B)** Compared to CNISO group, the changes was detected in NISO group. **(C,D)** Plot of unconstrained principal coordinate analyses based on weighted and unweighted UniFrac distances. NotSig, not significant.

Volcano plot was performed to identify OTUs associated with community separation between different groups with *P*-value cutoff of 0.05. Taking OTU abundance from CISO group as a control, there was little difference between the number of enriched OTUs and that of depleted OTUs (105 vs. 155) (Figure 3A). However, using OTU abundance from CNISO group as a control, the NISO group enriched many OTUs while depleting more OTUs at the same time (266 vs. 493) (Figure 3B). This result also demonstrated that bacterial richness in NISO group was lesser than that in CNISO group.

The overall structural changes of intestinal microbiota were then analyzed using unconstrained principal coordinate analyses (PCoA) based on weighted (Figure 3C) and unweighted (Figure 3D) UniFrac distances. The UniFrac distance is built on the basis of taxonomic relatedness, where the unweighted UniFrac metric only considers taxa exist or not whereas the weighted UniFrac also takes into account abundance of taxa. The first two dimensions of the PCoA score plot in Figure 3D described the unweighted UniFrac distances between different groups. Each point represented the microbial community of one sample. The PCoA plot displayed that microbial community of NISO group exhibited a significant shift along the positive direction of the first axis explaining 40.19% of the total variability and all samples in NISO group were observably separated from the other three groups. In addition, there was no significant difference between the groups of CISO and ISO. Meanwhile, weighted UniFrac analysis (Figure 3C) showed a similar changes of microbial community among these groups.

### Gut Microbial Composition at Different Taxonomical Levels

**FIGURE 4:**
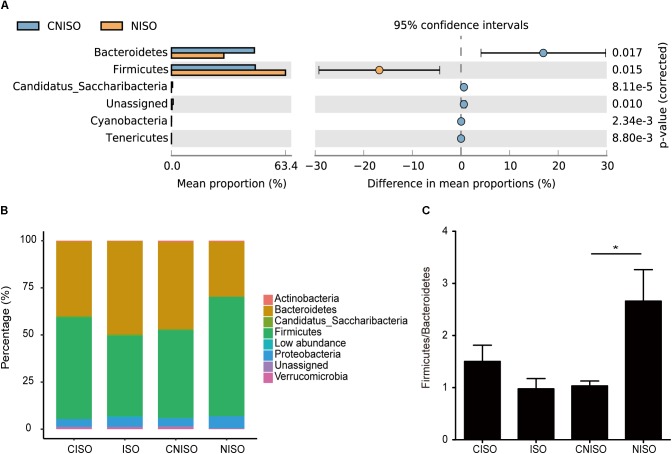
The differences of relative abundance of intestinal microbiota among four different groups at phylum level. **(A)** Welch’s *t*-test was used to compare the differences between the groups of CNISO and NISO. **(B)** The bar plot showed the percentages of intestinal microbiota communities in four groups. **(C)** The ratio of Firmicutes/Bacteroidetes in four groups. Data are presented as the mean ± SEM. ^∗^*P* < 0.05.

**FIGURE 5:**
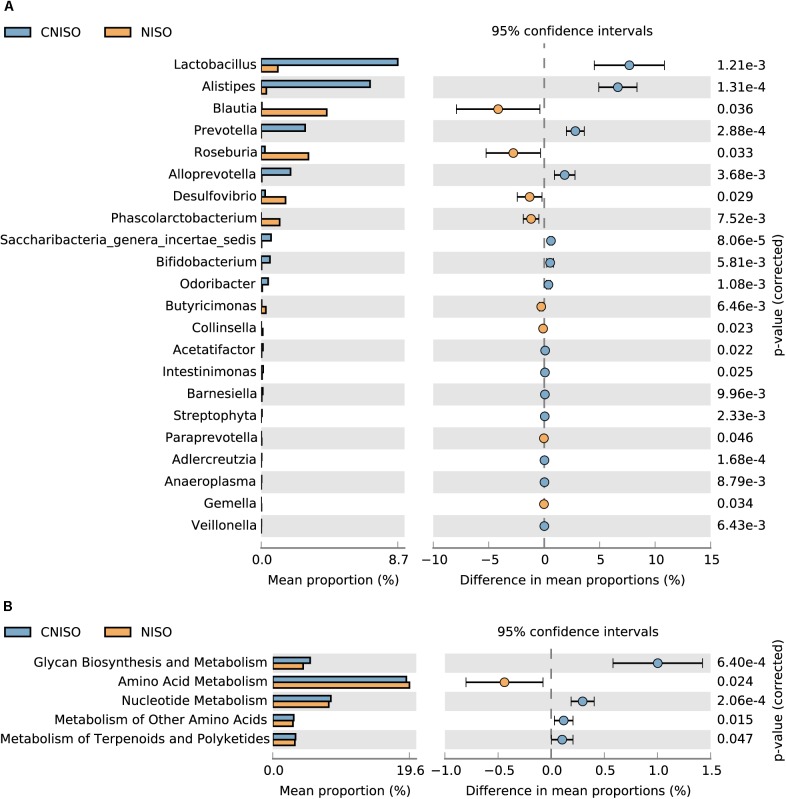
Differences in genus and Metagenomics levels between the groups of CNISO and NISO. **(A)** The differences of relative abundance of intestinal microbiota between the groups of CNISO and NISO at genus level. **(B)** Metagenomics data map related to metabolism between the groups of CNISO and NISO based on KEGG gene classification for feces samples at level-2 KO entries.

In the following work, taxon-based analysis also revealed that gut microbial compositions were shifted under the effect of non-isoflavones treatment. Ten phyla, which contained Firmicutes, Bacteroidetes, Proteobacteria, Actinobacteria, Candidatus Saccharibacteria, Cyanobacteria, Tenericutes, Deferribacteres, Lentisphaerae and Verrucomicrobia, were identified. Thereinto, Firmicutes, Bacteroidetes and Proteobacteria were the most primary phyla accounting for > 94% of the microbial compositions in all samples (Figure 4B). There was no significant difference between the groups of CISO and ISO at this level, whereas the relative abundance of five known phyla was changed obviously between the groups of CNISO and NISO (Figure 4A). Compared with CNISO group, the relative abundance of Firmicutes was increased and that of Bacteroidetes was decreased in samples of NISO group so that the ratio of Firmicutes to Bacteroidetes was increased after isoflavones deficiency (Figure 4C). Meanwhile, the proportions of Candidatus Saccharibacteria, Cyanobacteria and Tenericutes were also vastly reduced in NISO group. The compositions of intestinal microbiota in four groups were analyzed at the genus level. With time elapsing, the proportion of Alistipes was changed dramatically between the groups of CISO and ISO. 66 bacteria genera were detected from all samples with 64 and 65 genera in CNISO and NISO group, respectively. Besides Alistipes, the relative abundances of 21 genera were changed significantly in NISO group compared with those in CNISO group (Figure 5A). Among these data, isoflavones deficiency showed enriching effects on the relative abundances of 8 genera (Blautia, Roseburia, Desulfovibrio, Phascolarctobacterium, Butyricimonas, Collinsella, Paraprevotella, and Gemella), while the relative abundances of 13 genera (Lactobacillus, Prevotella, Alloprevotella, Saccharibacteria genera incertae sedis, Bifidobacterium, Odoribacter, Acetatifactor, Intestinimonas, Barnesiella, Streptophyta, Adlercreutzia, Anaeroplasma and Veillonella) were reduced in NISO group. Taken together, these results suggest that the bacterial ecosystem of feces in rats fed with non-isoflavones diet has changed compared with the feces in rats fed with normal chow diet.

### Functional Metagenome Prediction

To assess the metabolic potential effects on different diets, the PICRUSt analysis, a algorithm to predict the functional composition of bacterial metagenome using 16S rRNA gene sequences, was applied based on Kyoto Encyclopedia of Genes and Genomes (KEGG) database. The NSTI value was evaluated the accuracy of these predictions. The average NSTI of 0.17 ± 0.01 was obtained for CNISO group and average NSTI of 0.14 ± 0.03 was obtained for NISO group. These low values could accurately predict the molecular functions of bacterial communities in different groups of rats. The result of significant difference analysis at level-2 KO entries related to metabolism was shown in Figure 5B. A total of 5 metabolic functions at level-2 were significantly regulated. Compared to CNISO group, glycan biosynthesis and metabolism, nucleotide metabolism, metabolism of other amino acids, metabolism of terpenoids and polyketides were markedly down-regulated, whereas amino acid metabolism was up-regulated notably. Therefore, changes in bacterial community produced by non-isoflavones diet may change the various bacterial metabolic functions.

## Discussion

### Impact of Diet on Pharmacokinetic Traits

Modern pharmacological studies have shown that isoflavones have several proven beneficial effects on human health including alleviation constipation through several different mechanisms (Kim et al., 2015). However, no study has focused on the effects of isoflavones on constipation from the perspective of gut microbiota. Based on our previous pharmacokinetic study of rats with different diets adapted for 1 week (Yao et al., 2018), we also utilized two diets for 1 week to find the influence of intestinal microbiota and host health and to explain pharmacokinetic characteristics. Taken together, we set the control group and the experimental group to exclude the changes in intestinal microbiota over time in the present study. Based on the phenomenon of increased glycoside exposure and decreased glycogen exposure due to isoflavone deficiency, we further explore the relationship between diet and disease, diet and intestinal bacteria as well as intestinal microbiota and traits of metabolism.

### Impact of Diet on Disease Phenotypes

In terms of behavioral evaluation, fecal pellet numbers, water content, and the intestinal transit rate are common parameters to evaluate the intestinal function. Low fecal pellet numbers, low water content and slow intestinal transit rate are symptoms of animal constipation (Wang et al., 2017). According to our experimental results, the experimental group after non-isoflavones diet intervention showed obvious symptoms of constipation by reducing the values of above three parameters, which we could observe directly. Meanwhile, patients with constipation are usually accompanied by abnormalities in the Gastrointestinal hormones, including the deficiency of SP, which is able to promote intestinal peristalsis (Xiong et al., 2014). Although VIP is a type of non-cholinergic inhibitory neuropeptide, the reduction of VIP concentration can affect the effective promotion of colon and lead to constipation (Giancola et al., 2017). Therefore, the decrease of SP and VIP concentrations in the serum of rats suggested the occurrence of constipation in experimental group after non-isoflavones diet intervention. The effect of non-isoflavones diet on symptoms of constipation was reflected by intuitive behavioral parameters and biochemical parameters.

### Regulation of Diet on Intestinal Microbiota

Some studies have focused on the effects of isoflavones on intestinal microbiota (Guadamuro et al., 2015; Cross et al., 2017). Isoflavones, as Phytoestrogens, are commonly used to ameliorate menopause-associated obesity and metabolic dysfunction (Hirose et al., 2016). Therefore, fecal samples from postmenopausal women are often used as subjects in the study of the relationship between isoflavones and intestinal microbiota (Guadamuro et al., 2015). However, we investigated the influence of isoflavones deficiency on the health of the intestine itself in the biochemical state of rats.

According to our results, both the diversity indexes and principal coordinate analysis showed that isoflavones deficiency induced the gut microbiota dysbiosis. Meanwhile, the relative abundance of Firmicutes and Bacteroidetes, main bacterial groups involved in the metabolism of undigested food, were increased and decreased, respectively. The ratio of Firmicutes to Bacteroidetes as an evaluation indicator of gastrointestinal health was increased and this phenomenon was usually associated with constipation and metabolic diseases. Additionally, Prevotella, belonging to Bacteroidetes, has the ability to encode multiple glycoside hydrolases (Purushe et al., 2010). It also had a decreased relative abundance after non-isoflavones diet intake, whereas Blautia had an increased relative abundance. The changes in the intestinal microbiota composition in the group with non-isoflavones diet intervention was similar to the previous research about constipation (Ren et al., 2017).

### Regulation of Enzyme-Mediated Intestinal Microbiota on Traits of Metabolism

Lactobacillus and Bifidobacterium, prominent among probiotics, are long thought to be the most common microbes in human and other animals intestinal microbiota and tightly related with good intestinal health. They provide a wide range of health promotion functions for the host, such as inhibition of pathogens, enhancement of barrier function, improvement of immunity and interaction with inflammation mediators (Wu et al., 2018). Several studies demonstrated that the mechanisms of Lactobacillus and Bifidobacterium against intestinal pathogens included reduction the pH level to restrain excessive reproduction of harmful bacteria, competing adhesion sites with pathogens and production of antimicrobial metabolites (Gibson et al., 2007; Vinusha et al., 2018). In this study, their relative abundances were decreased after non-isoflavones diet intake. This result was consistent with the intestinal microbiota composition of chronic constipation patients (Khalif et al., 2005). However, the relative abundance of Lactobacillus and Bifidobacterium exhibited increasing trends in the gut of the constipated obese adolescents compared with non-constipated obese adolescents without statistically significant (Zhu et al., 2014). Furthermore, some studies indicated that Lactobacillus could produce extracellular β-glucosidase which contribute to biotransform glycosides into aglycons to improve their bioavailabilities and exerted the bioactivities consequently (Rekha and Vijayalakshmi, 2010; Yeo and Liong, 2011). Thus, we could explain the differences in plasma exposure of glycosides and aglycones under different dietary conditions from microcosmic angle of enzyme activity (Yao et al., 2018). Meanwhile, the gut functional metagenome prediction of the experimental group was performed by PICRUSt. We screened out metabolic functions with significant differences at level-2 KO entries using Welch’s *t*-test and found that glycan biosynthesis and metabolism was down-regulated after non-isoflavones diet intake to explain the pharmacokinetic characteristics of increased glycosides exposure and decreased aglycones exposure in non-isoflavones diet compared to the normal chow diet from a macroscopic perspective.

In our study, non-isoflavones diet intake increased the relative abundance of Desulfovibrio. The genus Desulfovibrio, a dominant group of Sulfate Reducing Bacteria (SRB), is a class of anaerobic bacteria which can reduce sulfate to produce H_2_S. Endogenous H_2_S can inhibit the oxidation of acid salts, affect the respiration of cells and induce apoptosis and chronic inflammation. It has been reported that Desulfovibrio is related to the occurrence and development of colon cancer, irritable bowel syndrome, metabolic syndrome and other diseases (Crispim et al., 2018). Hence, Isoflavones are essential in the diet.

In order to verify the regulation of enzyme-mediated intestinal microbiota on traits of metabolism, rats were gavage with the high-dose total isoflavones extract (0.21g/kg) for different weeks to observe the effect of total isoflavones on pharmacokinetic traits of iridoid glycosides. It was found that not only the exposure of glycosides but also that of aglycons were increased, while the exposure of sulfate conjugates was decreased in blank group compared with the experimental group rats. The biotransformation of glycosides to aglycons is due to the hydrolysis of β-glucosidase. The process of producing sulfate conjugates by combining aglycons with the sulfate is owing to the sulfotransferase, which can play a role in detoxification. Several studies (Kamaladevi et al., 2016; Ren et al., 2018) have shown that oral Lactobacillus can modulate phase-II metabolic enzymes, such as sulfotransferase, UDP-glucuronosyltransferases, etc. Therefore, we speculate that Lactobacillus in the intestinal tract may affect sulfotransferase. In our results, the abundance of Lactobacillus in the NISO group was reduced compared with the CNISO group, which could be inferred that the Lactobacillus abundance in the blank group was lower than that in the experimental group. Thus, the activities of β-glucosidase and sulfotransferase were lower in the blank group, which hinder the biotransformation of glycosides to aglycons and aglycons to sulfate conjugates. Furthermore, Desulfovibrio can reduce sulfate to sulfide. The key enzymes in this process are dissimilatory adenosine 5′-phosphosulfate reductase (AprAB) and sulfite reductase (Duarte et al., 2016). The abundance of Desulfovibrio in the NISO group was higher than that in the CNISO group, which could be inferred that the Desulfovibrio abundance in the blank group was higher than that in the experimental group. Thus, the activities of AprAB and/or sulfite reductase in the blank group were higher than those in the experimental group to promote the consumption of sulfate conjugates. Overall, the production and consumption of active metabolites were affected by enzyme-mediated intestinal microbiota to play an important role in synergy and reduction of toxicity.

### New Strategy of DIET

**FIGURE 6:**
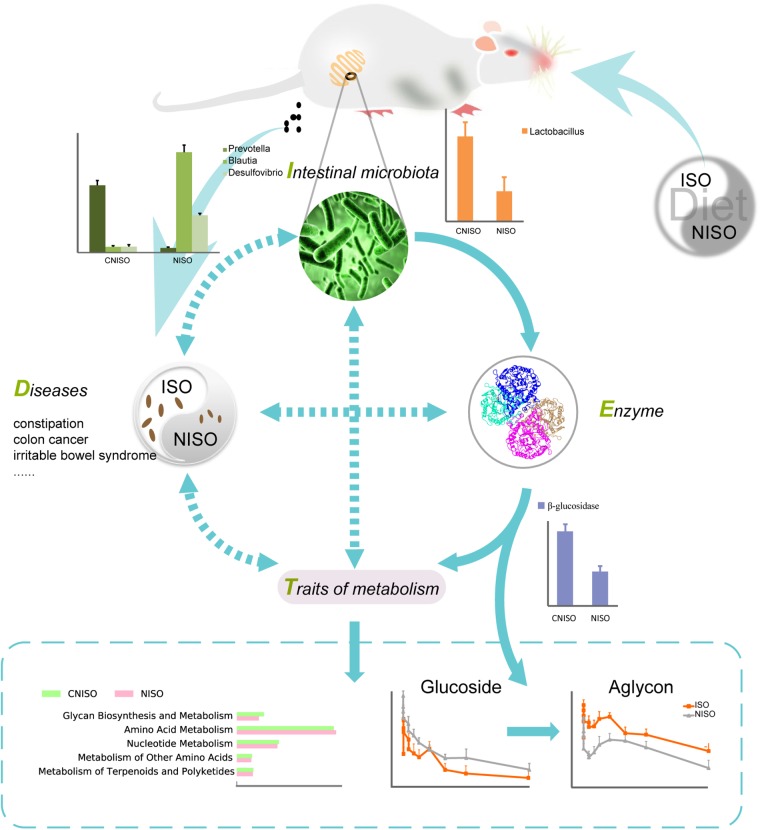
The interactions and underlying mechanisms of our new strategy DIET through regulation intestinal bacteria. The solid arrows show clear action processes. The dotted arrows indicates that the mechanism is blurry.

Recent studies have shown the important relationship among dietary, intestinal microbiota and human health (Gutiérrez-Díaz et al., 2016; Moreno-Indias et al., 2016). To the best of our knowledge, this is the first time using 16S rRNA gene sequencing to characterize the intestinal microbiota of healthy rats fed non-isoflavones diet in constipation perspective. By decreasing the amount of Prevotella, Lactobacillus and Bifidobacterium and enriching the abundance of Blautia and Desulfovibrio, non-isoflavones diet can induce dysbacteriosis to cause constipation and other adverse effects on intestinal health. In addition, predicted metagenomics indicated that the pathway of glycan biosynthesis and metabolism was markedly down-regulated after non-isoflavones diet intake. Overall, alteration of intestinal microbiota can trigger changes in enzyme activity, and then lead to changes in concentrations of active metabolites which closely associated with the prevention and alleviation of constipation (Figure 6). Accordingly, down-regulation of good bacteria due to isoflavones deficiency make it difficult to metabolize and detoxicate, while up-regulation of bad bacteria can produce harmful substances that aggravate the disease by controlling the activity of related enzyme. It means that a new strategy named as “DIET” can provide a reference for further study of the mechanism in regulation of intestinal bacteria-mediated diet on diseases.

## Author Contributions

JL, RC, XZ, and ZW performed the experiments. JL and RC analyzed the sequence data. JW gave suggestions for writing. TZ and JL drafted the manuscript. TZ designed the whole research and reviewed the final manuscript. All the authors have read and approved the final version.

## Conflict of Interest Statement

The authors declare that the research was conducted in the absence of any commercial or financial relationships that could be construed as a potential conflict of interest.

## Footnotes

1 http://www.drive5.com/usearch

2 http://rdp.cme.msu.edu

3 http://huttenhower.sph.harvard.edu/galaxy
