# Supplementary material for: Non-isoflavones Diet Incurred Metabolic Modifications Induced by Constipation in Rats via Targeting Gut Microbiota
Source: Front Microbiol. 2018 Dec 4;9:3002. doi: 10.3389/fmicb.2018.03002 (PMC6288237; doi:10.3389/fmicb.2018.03002)
Supplement: Supplementary file 1 [file Table_1.DOCX]

Supplementary Material

Non-isoflavones Diet Incurred Consitipation in Rats via Triggering Abnormal Changes with Gut Microbiota

Jialin Liu, Zhongzhao Wang, Yiyang Li, Sai Liu, Jun Wen and Tingting Zhou*

School of Pharmacy, Second Military Medical University, Shanghai, China

* Correspondence:

Tingting Zhou

tingting_zoo@163.com

**
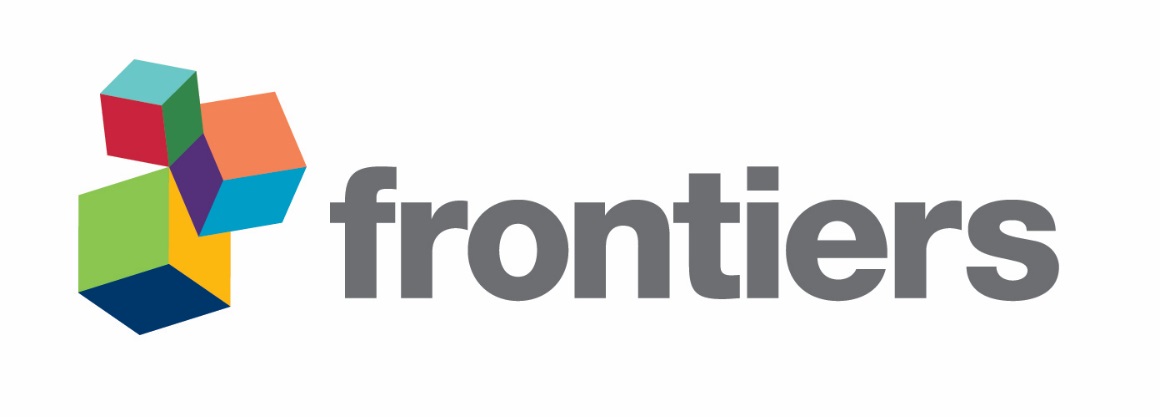
**

# Supplementary Table

Supplementary Table 1. Serum Substance P (SP) and Vasoactive Intestinal Peptide (VIP) concentrations of rats fed experimental diets compared with the ISO group. Data are presented as the means ± SEM. **P < 0.01.

|  | ISO | NISO |
| --- | --- | --- |
| SP (ng/mL) | 2.1 ± 0.13 | 1.6 ± 0.09** |
| VIP (pg/mL) | 90.70 ± 4.06 | 75.81 ± 2.13** |
